# Examination of Oral Microbiota Diversity in Adults and Older Adults as an Approach to Prevent Spread of Risk Factors for Human Infections

**DOI:** 10.1155/2017/8106491

**Published:** 2017-09-10

**Authors:** Paweł J. Zawadzki, Konrad Perkowski, Marcin Padzik, Elżbieta Mierzwińska-Nastalska, Jacek P. Szaflik, David Bruce Conn, Lidia Chomicz

**Affiliations:** ^1^Clinic of Cranio-Maxillo-Facial and Oral Surgery and Implantology, Medical University of Warsaw, 4 Lindleya Str., 02-005 Warsaw, Poland; ^2^Department of Orthodontics, Medical University of Warsaw, 59 Nowogrodzka Str., 02-006 Warsaw, Poland; ^3^Department of Medical Biology, Medical University of Warsaw, 73 Nowogrodzka Str., 02-018 Warsaw, Poland; ^4^Department of Prosthodontics, Medical University of Warsaw, 59 Nowogrodzka Str., 02-006 Warsaw, Poland; ^5^Department of Ophthalmology, SPKSO Ophthalmic Hospital, Medical University of Warsaw, 13 Sierakowskiego Str., 03-709 Warsaw, Poland; ^6^Department of Invertebrate Zoology, Museum of Comparative Zoology, Harvard University, Cambridge, MA 02138, USA; ^7^One Health Center, Berry College, School of Mathematical and Natural Sciences, Mount Berry, GA 30149-5036, USA

## Abstract

The oral cavity environment may be colonized by polymicrobial communities with complex, poorly known interrelations. The aim of this study was to determine oral microbiota diversity in order to prevent the spread of infectious microorganisms that are risk factors for human health complications in patients requiring treatment due to various disabilities. The study examined Polish adults aged between 40 and 70 years; parasitological, microbiological, and mycological data collected before treatment were analyzed. The diversity of oral microbiota, including relatively high prevalences of some opportunistic, potentially pathogenic strains of bacteria, protozoans, and fungi detected in the patients analyzed, may result in increasing risk of disseminated infections from the oral cavity to neighboring structures and other organs. Increasing ageing of human populations is noted in recent decades in many countries, including Poland. The growing number of older adults with different oral health disabilities, who are more prone to development of oral and systemic pathology, is an increasing medical problem. Results of this retrospective study showed the urgent need to pay more attention to the pretreatment examination of components of the oral microbiome, especially to the strains, which are etiological agents of human opportunistic infections and are particularly dangerous for older adults.

## 1. Introduction

In recent decades, oral microbial communities have often been the subject of studies regarding the health and sickness in various human populations; they were undertaken using different methodologies, including recent advances in sequencing technology [1–6].

The environment of the oral cavity is an open, dynamic system, with diverse conditions of specific, specialized surfaces of soft tissues as well as hard structures. The studies on this system have been focused primarily on oral microbiota as factors associated with oral diseases: dental caries and periodontitis. In particular, special emphasis has been placed on the important role of Gram-positive bacteria of the* Streptococcus “viridians”* group, which are considered to be resident inhabitants participating in the development of biofilm [4, 7–9]. Early colonizers inhabit the oral cavity in an initial postnatal stage; as microbes grow, with development of teeth, they modify the local environment and facilitate colonization by more microorganisms. The oral biofilm is subsequently formed on surfaces of mucous membranes and teeth, until they ultimately are structurally and functionally organized into polymicrobial communities. Among these,* Scardovia wiggsiae *strains are etiological agents of early childhood caries, while, in adults,* Streptococcus oralis*,* S. salivarius*, and* S. mitis* are known as causative agents of caries [10, 11]. Results of more recent studies have expanded the list of cariogenic bacteria. For example, it was shown for the first time by Belda-Ferre et al. [12] in their metagenome description of the human oral cavity under health and diseased conditions—with a focus on supragingival dental plaque and cavities—that a synergistic effect is likely; thus, it was confirmed that caries is a polymicrobial disease [12]. Numerous studies on oral microbiota have emphasized the etiological agents of periodontitis in adults; among others, a role has been confirmed for bacteria from the genera* Actinobacillus* and* Campylobacter*, and also* Porphyromonas gingivalis*,* Tannerella forsythia*, and* Treponema denticola* [2, 13]. The suggestion that “*Porphyromonas gingivalis* may act as a classic keystone species assisting in the transformation of the oral microbiota from an otherwise benign state to a pathogenic state via dysbiosis, elevated community virulence, and inflammation” was discussed by Lunsford et al. [6].

The important subject of several investigations has been the associations between oral cavity microbiota species composition and diseases of the masticatory system, in reference to various local, congenital, structural, metabolic, and general diseases [2–4, 8, 10, 13–15]; among these, certain studies revealed that oral microbiota can cause hospital-acquired infections and general health problems. Investigations of oral cavity microbiota in terms of their connections with selected disorders in patients of different populations were also undertaken by the authors of the current study [16–18].

In Poland, since dental caries and periodontitis are the major oral health problems, most of the research pertains to bacteria as etiological factors of these important social diseases.

Relationships between various groups of microorganisms, including protozoans, fungi, and bacteria that can colonize the oral cavity in conjunction with the human clinical condition, have not been extensively studied. The results of our previous interdisciplinary studies revealed species differences in oral microbial communities in patients with masticatory system disorders and with certain systemic diseases. In these, the immunological condition of the patients and the therapeutic procedures had different degrees of influence on the changes in oral microbiota [16–20].

In our current analysis of oral microbiota, our comparative assessment pertained to adults and older adults, the population groups that have been increasing in recent decades in Poland and other countries and who required routine and specific treatment due to various disabilities.

In the present retrospective study, the diversity in species composition of the oral cavity microbiome was monitored to explore the possibility of a preventive approach to slow spread of infectious microbiota as risk factors of human infections.

## 2. Materials and Methods

For the retrospective comparative study we examined data on 85 Polish adults and older adults, of both sexes, aged between 40 and 70 years who received routine and specific treatment in Clinics of the Medical University of Warsaw (2006–2014). The patients were classified into 3 age groups: Group I involved thirty persons from 41 to 50 years; Group II involved thirty persons 51 to 60 years, and Group III included twenty-five persons from 61 to 70 years.

Patients were examined clinically; particularly, periodontal status (the presence of inflammation), dental caries, and restorative dental treatment were assessed.

The criteria for inclusion in this study were age over 40 years, generally healthy patients, no or few missing teeth, not restored prosthetically, no symptoms of periodontitis, and no more than 5 teeth with gingivitis. Data of patients with systemic diseases and who were treated prosthetically were excluded from this analysis.

For qualitative research and microorganism species identification, oral swabs were collected (avoiding contamination of samples with extrinsic components, e.g., before dental treatment or meal) and performed according to a previously applied procedure [16, 17, 21]. Swabs from each patients, taken directly from ten sites of supragingival dental plaques, marginal gingiva, and dental pockets, were placed in sterile tubes containing physiological salt solution, at pH 6.8 and 36°C. The biofilm materials were used for preparation of wet and permanent slides for light microscopic examinations. The Giemsa and trichrome-stained slides were examined for identification of protozoans and preliminary determination of Gram-positive and Gram-negative bacteria strains. Bacteriological microscopic and* in vitro* standard culture techniques were also applied to isolate bacterial strains. Samples of the material were grown aerobically on bacteriological agar and on agar with 5% defibrinated sheep blood and then tested for further specific determination. MacConkey's medium was applied to isolate bacteria of Enterobacteriaceae, and Chapman's plate growth medium for detection and isolation of staphylococci. The material from oral swabs was also used for mycological laboratory tests. Sabouraud medium (bioMerieux) was applied for growth of fungi at 30°C;* Candida* species were identified in the samples by chromogenic medium Chromagar-Candida BBL (Becton Dickinson).

The species composition of microbiota detected in oral cavities and the prevalence of particular species were compared between particular patient groups and analyzed statistically. *F*-Fisher and HSD Tukey tests for an assessment of statistical significance (*p* ≤ 0.05) and Statistica software were applied.

## 3. Results

The retrospective study of clinical examination revealed certain differences in oral cavity conditions of analyzed patients from particular age groups. Restorative dental treatment was conducted in all patients but loose teeth, gingival bleeding, and limited gingival inflammations were noted more frequently in the oldest persons.

Data from microscopic examinations of samples deriving from oral cavity swabs, cultures, and* in vitro* tests showed the presence of various microorganisms belonging to different genera, species, and strains of bacteria, protozoans, and fungi in patient groups analyzed. The potentially pathogenic microbiota identified in oral cavities of patients of particular age groups are listed in Table 1.

In some fresh specimens assessed microscopically, live protozoans (flagellates and amoebae) were detected between the epithelial cells and white and red blood cells; moreover, in some samples, amoebic cysts were found. Flagellates, based on their morphology, were identified as* Trichomonas tenax*; these trichomonads moved quickly, each with the median rod axostyle; 4 free flagella, the fifth flagellum associated with undulating membrane, were 10–15 *μ*m long and 5–11 *μ*m wide, with vacuoles that contained multiplying bacteria. The moderately active, amoebic trophozoites, with changing-shape, characteristic ectoplasm, and blunt pseudopodia, were identified as* Entamoeba gingivalis*; these measured 5–15 *μ*m × 20–35 *μ*m and were detected mainly in dental pockets. Vacuoles of the amoebae contained bacteria and erythrocytes.

The comparative assessment of analyzed data showed that occurrence of flagellates and oral amoebae varied in relation to patient age. Statistically significant differences (*p* < 0.05) occurred in the patients of Groups 1 and 3.* T. tenax* was detected with significantly higher frequency than* E. gingivalis* in persons of Group I, 41–50 years (prevalence 30% and 10%, resp.); contrary to this, trichomonads occurred with lower frequency than* E. gingivalis* in the oldest patients of Group III, 61–70 years (prevalence 8% and 24%, resp.).

In some periodontal samples from four patients (13.3%) of Group II, double-walled cysts of 8–25 *μ*m diameter were found and identified as cysts of free-living, facultative parasitic* Acanthamoeba* spp. These findings of Protista were verified by stained slide examinations. After excystation, some of the amoeba trophozoites remained alive for several days in physiological salt solution.

In the material collected from patients and used for Chromagar-Candida BBL cultivation, various strains of yeast-like fungi from the* Candida* genus, predominantly of* Candida albicans* group, were isolated and identified. These fungi were detected in patients from all groups analyzed; the highest prevalence of* C. albicans* strains, 30%, occurred in Group II of patients from 51 to 60 years.

Bacteria of the* Streptococcus viridans* group and* Moraxella *genus were identified in all assessed patients; data regarding these Gram-positive bacterial strains, considered as typical inhabitants of the human oral cavity, were not analyzed in this study.

The assessment of microscopic and* in vitro* cultured samples of oral swabs showed presence of certain opportunistic bacteria belonging to five Gram-positive and six Gram-negative bacteria species on the surface of the mucous membrane of the periodontium and dental plaques and in dental pockets. Among those Gram-positive,* Enterococcus faecalis* bacteria were detected in all groups, with the highest prevalence being 16.6% in older patients of Group II. Potentially pathogenic staphylococci* Staphylococcus epidermidis* and* Staphylococcus aureus* occurred in the oral cavity of some patients of Groups I, II, and III.* Micrococcus luteus* and* Enterococcus faecium* were sporadically found only in persons from Group I.

Among Gram-negative bacteria,* Escherichia coli* strains occurred in all patient groups with various prevalence.* Pseudomonas aeruginosa* was isolated from the oral cavities of patients of Group I and Group II. Some strains of* Klebsiella oxytoca*,* K. pneumonia*, and* Acinetobacter baumannii* were detected only in Group I while* Pantoea agglomerans* was found only in patients of Group II. No statistically significant differences in frequency of particular microorganisms between male and female patients were revealed.

**Figure 1:**
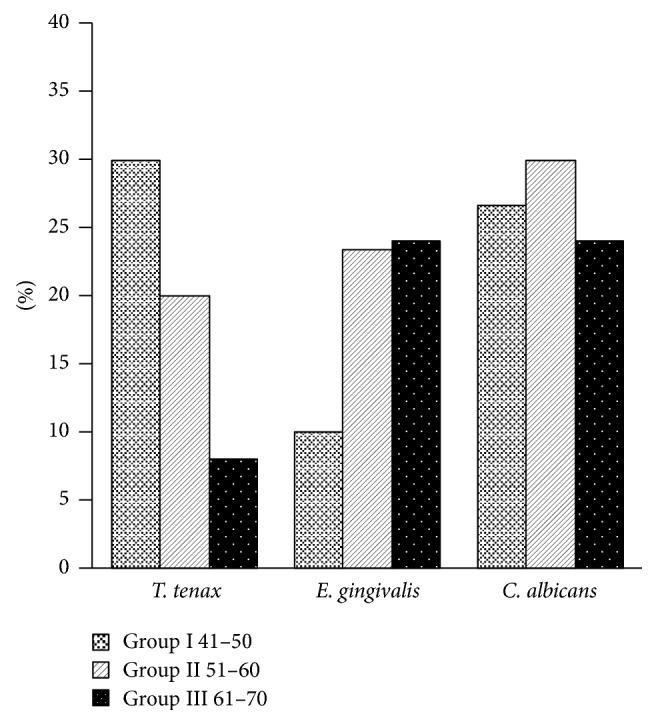
The comparison of prevalence of trichomonads, oral amoeba, and yeast-like fungi detected in oral cavities of particular patients from all groups analyzed.

**Figure 2:**
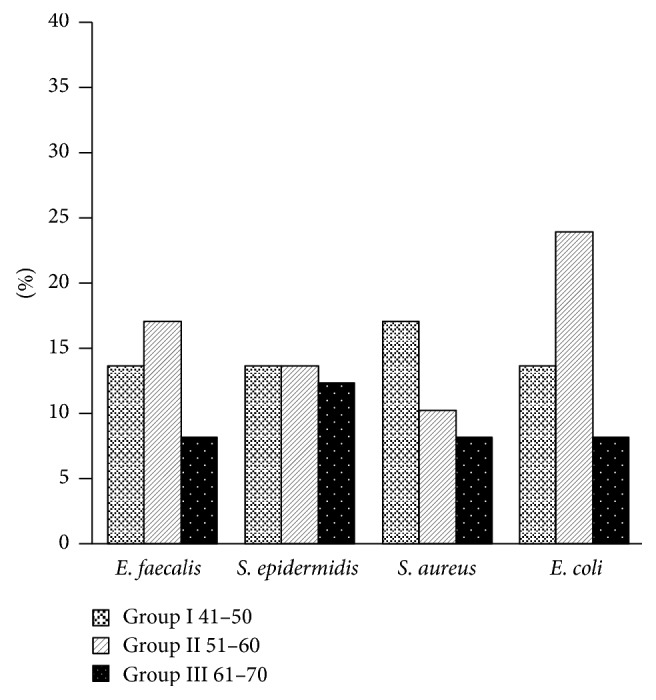
The comparison of prevalence of selected oral microbiota—the Gram-positive and Gram-negative bacteria revealed in all patient groups.

The comparison of prevalences of microbiota that have been detected in oral cavities of all patient groups is presented in Figures 1 and 2.

## 4. Discussion

Oral cavity microbiota are considered to be of heterogeneous origin and can include various endo- and exogenous species [1, 2, 10, 12–14]. The technological development, advances in amplification and sequencing tools, and large-scale genome analysis increase possibilities for diagnostics of species uncultivable at present; this can impact the knowledge about microorganisms that can colonize the oral cavity [6, 10, 13]. “Studies involving the oral microbiota metagenomic projects like the HMP” (Human Microbiome Project) “have confirmed that the oral cavity is one of the most taxonomically diverse body sites…” as has been emphasized by Lunsford et al. [6] from the National Institute of Dental and Craniofacial Research, National Institutes of Health, Bethesda, MD, USA.

Our recent study showed that different microorganisms occurred in the material from supragingival dental plaque, a surface of marginal gingival, and dental pockets examined directly and with use of appropriate selective culture media. In the polymicrobial communities, some infectious species were identified as protists, fungi, and bacteria.

It is emphasized in literature that, in generally healthy persons, complex interrelations occur between multilayer components of biofilm, particular species of oral microbiota, and the host organism. This results in relatively constant composition of the oral cavity microbiome because the human immunological system influences the oral environment stability by some inhibition of multiplication activity of microbiota, which remain in labile homeostasis [4, 7, 8, 11]. Many biotic and abiotic factors that can alter this homeostasis may influence the multispecies microbial communities and increase the number of certain endogenous bacteria. This can also lead to a colonization of the oral cavity by exogenous species, including those that are potentially pathogenic.

Results of our comparative analysis regarding monitoring of the frequencies of microbiota components, detected in various age groups, showed clear differences in the prevalences of particular strains.

Protists species, such as flagellates and amoebae, are not so often taken into consideration in clinical researches, but can have a pathogenic impact on oral structures. Among others, the fibronectin-like protein and collagenolytic effect of trichomonads and also oral amoeba activity in inflammatory gingivitis and in pathogenic dental pockets are underscored [16, 22–25].

Oral protozoans are rarely found in children; they were more frequent in older persons. High prevalence and large number of microorganisms were found in persons showing pathological changes in their oral cavities: patients with systemic diseases and with decreased resistance connected with congenital disease, as well as in patients under chronic immunosuppression. For example, in 40–50-year-old patients with somatic and mental retardation connected with epilepsy or Down syndrome, the oral amoebae and trichomonads occurred with prevalence from 30% to 60%. These and other observations lead to a conclusion of an opportunistic nature of the protozoans [16–19, 25].

In the last few decades, the percentage of older adult Polish citizens with decreased masticatory function significantly increased [26]. Relatively high prevalences of oral trichomonads and* E. gingivalis* in the present study, and, also the detection of* Acanthamoeba* spp. cysts in older adult patients, are in agreement with several previous findings [8, 16–18, 24, 27]. Various strains of* Acanthamoeba* sp. are worldwide amphizoic amoebae that may cause serious human health threats as etiological agents of granulomatous amoebic encephalitis and vision-threatening* Acanthamoeba* keratitis. The amoebae were isolated from the hospital environment, among others, as contaminants of surgical instruments and the dental irrigation system. It is considered that ontogenetic-depending factors influence the oral protozoans that are agents predisposing to local inflammations and a real threat of disseminated infections [19, 22–25, 27–31].

Yeast-like fungi from the* Candida* genus, predominantly of the* Candida albicans* group, isolated and identified from the oral cavity of all age groups analyzed, occur commonly in the human environment. The fungi colonize humans in early stages of ontogenetic development; they may infect different host tissues and organs also later, via skin, the damaged mucous membranes, inhalation, or ingestion. Different factors favour the infection of the oral cavity by fungi and development of candidosis, namely, improper oral cavity hygiene, micro-damage, presence of foreign bodies, and acrylic dental prosthesis attached to the mucous membrane and affecting its surface. Recently, it has been considered that yeast-like fungi are particularly prone to produce biofilms [8, 32] and that this ability is an important virulence factor of these pathogens.* Candida *spp. strains have been detected in oral cavities in up to 70% of human populations. According to literature data [5, 8, 32–34], candidosis occurs in patients with congenital malformation, under immune-suppression, after a surgery, and under antibiotic and cytostatic therapies. It is known that colonization of the oral cavity and other organs with yeast-like fungi constitutes a medical problem among patients during invasive procedures, with impaired immunological defense mechanisms. The disease usually develops as an endogenous opportunistic infection; compounds secreted by the fungal cells my induce lowering of the immunological response and thus sustain a threat of disseminated infection also in immune-competent persons.

Our comparative qualitative analysis showed the occurrence of various potentially infectious bacterial species detected on the marginal gingiva, supragingival dental plaque, and dental pockets. Some differences in the prevalence of the strains were noted in the oral cavities of particular patient groups. Among Gram-positive bacteria,* Enterococcus faecalis* strains that were detected in all groups of analyzed patients are common in the human intestine. The bacteria were isolated from the mouth of healthy patients and those with masticatory system disorders that required surgical treatment [17, 18]; they may act as opportunistic agents of hospital-acquired diseases, including stomatopathy, pneumonia, and nosocomial urinary tract infections. The latter are especially dangerous for immune-compromised and elderly persons.

Potentially pathogenic staphylococci occurred in some patients of all groups. The habitat of* Staphylococcus epidermidis*, the member of the group of coagulase-negative staphylococci, is skin and mucous membranes; however, the species may be causative agent of local oral inflammations, for example, gingivitis and endodontic infections [20, 21]. The most important factor in pathogenesis of these staphylococcal infections is the formation of bacterial biofilm; during the process, bacteria adhere to the surface to be colonized and then accumulate into multicellular and multilayered structure; cells liberated from the biofilm are able to colonize additional body sites [8, 35, 36].* S. epidermidis* is believed as one of important opportunistic bacteria related to hospital-acquired infections, most frequent cause of nosocomial blood stream infections [21, 35]. Important complications may be generated by* Staphylococcus aureus* strains that also participate in the biofilm formation; biomaterial-associated infections are most frequently caused by this bacteria species [8, 35, 37]. Disseminations of the pathogens, particularly MRSA-methicillin-resistant strains, to different distant sites result in difficult-to-treat diseases, for example, those associated with surgical procedures, pneumonia, and endocarditis [17, 18, 35, 37]. Among Gram-negative bacteria,* Escherichia coli* and* Pseudomonas aeruginosa* are known for their epidemiological role; the bacteria are particularly dangerous for immune-compromised persons; they were also detected in oral cavities of patients with systemic diseases [16, 20]. Among* E. coli* rods, enteropathogenic, enterotoxic, enterohemorrhagic strains may be the causative agents of nosocomial infections and sepsis. It is noteworthy that* P. aeruginosa* bacteria are particularly prone to produce oral cavity biofilms [8].

Currently, about half of bacterial species that can inhabit the human oral cavity are culturable. Advances in molecular diagnostic tests are very helpful in determination of nonculturable species and thus greatly expand the possibilities of identification of microbiome components of the human oral cavity. Nevertheless, recently the technical and economic limitations, in addition to other factors, make culture methods still the basis in every laboratory research study and in clinical applications as important parts of the baseline diagnostics and adequate therapy. Results of our retrospective study showed that in oral cavities of Polish adults and older adults many different microorganisms potentially pathogenic for humans may occur. Investigations of composition diversity in the oral microorganisms may be useful for preventive approaches to the development of human general and nosocomial diseases and for treating elderly patients.

## 5. Conclusions

Among many factors that influence the oral environment, some are age dependent; for example, an immunogenic ability may change complex interrelations between particular species of oral microbiota and host organism during ontogenesis in generally healthy persons. Increasing ageing of human populations has been noted in recent decades in many countries, including Poland. Although increasing life expectancy is a positive phenomenon, it results in a growing number of older patients with different oral health problems, often requiring surgical treatment. The patients, even without systemic diseases, are more prone to development of oral and systemic pathology infections because of their often lower immunological adaptability, resulting from the ontogenetic factor—advanced age. It should be taken into account that the oral cavity may act as a reservoir of infectious microbiota. Results of this retrospective study showed the urgent need to give more attention to diversity in the oral microbiome; especially important are advances in knowledge about the threat of disseminated protozoan, fungal, or bacterial infections from oral cavity to neighboring structures and other organs.

The pretreatment examination of oral cavity microbiota may be helpful in a preventive approach to the spread of infectious microorganisms, which may be etiological agents of human opportunistic infections and risk factors for treatment complications, particularly dangerous for older adults.

## Figures and Tables

**Table 1:** Potentially pathogenic microbiota identified in oral cavities of patients of particular age groups.

Group and age of patients	Microbiota identified in oral cavities		
	Protista	Fungi	Bacteria
I 41–50	*Trichomonas. tenax* *Entamoeba. gingivalis*	*Candida albicans* *Candida glabrata* *Candida* spp.	Gram-positive bacteria strains *Enterococcus faecalis* *Enterococcus faecium* *Staphylococcus epidermidis* *Staphylococcus aureus* *Micrococcus luteus* Gram-negative bacteria strains Enterobacteriaceae*:* *Escherichia coli* *Klebsiella oxytoca* *Klebsiella pneumoniae* Non-Enterobacteriaceae*:* *Acinetobacter baumannii* *Pseudomonas aeruginosa*

II 51–60	*Trichomonas. tenax* *Entamoeba. gingivalis* *Acanthamoeba *sp.	*Candida albicans* *Candida* spp.	Gram-positive bacteria strains *Enterococcus faecalis* *Staphylococcus epidermidis* *Staphylococcus aureus* Gram-negative bacteria strains Enterobacteriaceae: *Pantoea agglomerans* *Escherichia coli* Non-Enterobacteriaceae *Pseudomonas aeruginosa*

III 61–70	*Trichomonas. tenax* *Entamoeba. gingivalis*	*Candida albicans* *Candida* spp.	Gram-positive bacteria strains *Enterococcus faecalis* *Staphylococcus epidermidis* Gram-negative bacteria strains Enterobacteriaceae*:* *Escherichia coli*
